# Pathogenicity and Immunogenicity of Recombinant Rabies Viruses Expressing the Lagos Bat Virus Matrix and Glycoprotein: Perspectives for a Pan-Lyssavirus Vaccine

**DOI:** 10.3390/tropicalmed2030037

**Published:** 2017-08-09

**Authors:** Joe Kgaladi, Milosz Faber, Bernhard Dietzschold, Louis H. Nel, Wanda Markotter

**Affiliations:** 1 Centre for Emerging and Zoonotic Diseases, National Institute for Communicable Diseases, National Health Laboratory Service, Sandringham 2193, South Africa; joe.kgaladi@gmail.com; 2 Department of Microbiology and Immunology, Thomas Jefferson University, Philadelphia, PA 19107, USA; milosz.faber@jefferson.edu (M.F.); bernhard.dietzschold@jefferson.edu (B.D.); 3 Department of Microbiology and Plant Pathology, Faculty of Natural and Agricultural Sciences, University of Pretoria, Pretoria 0001, South Africa; louis.nel@up.ac.za; 4 Centre for Viral Zoonoses, Department of Medical Virology, Faculty of Health Sciences, University of Pretoria, Pretoria 0001, South Africa

**Keywords:** rabies, vaccines, Africa, lyssavirus, pathogenesis, recombinant viruses

## Abstract

Lagos bat virus (LBV) is a phylogroup II lyssavirus exclusively found in Africa. Previous studies indicated that this virus is lethal to mice after intracranial and intramuscular inoculation. The antigenic composition of LBV differs substantially from that of rabies virus (RABV) and current rabies vaccines do not provide cross protection against phylogroup II lyssaviruses. To investigate the potential role of the LBV matrix protein (M) and glycoprotein (G) in pathogenesis, reverse genetics technology was used to construct recombinant viruses. The genes encoding the glycoprotein, or the matrix and glycoprotein of the attenuated RABV strain SPBN, were replaced with those of LBV resulting in SPBN-LBVG and SPBN-LBVM-LBVG, respectively. To evaluate the immunogenicity of the LBV G, the recombinant RABV SPBNGAS-LBVG-GAS was constructed with the LBV G inserted between two mutated RABV G genes (termed GAS). All the recombinant viruses were lethal to mice after intracranial (i.c.) inoculation although the pathogenicity of SPBNGAS-LBVG-GAS was lower compared to the other recombinant viruses. Following intramuscular (i.m.) inoculation, only SPBN-LBVM-LBVG was lethal to mice, indicating that both the M and G of LBV play a role in the pathogenesis. Most interestingly, serum collected from mice that were inoculated i.m. with SPBNGAS-LBVG-GAS neutralized phylogroup I and II lyssaviruses including RABV, Duvenhage virus (DUVV), LBV, and Mokola virus (MOKV), indicating that this recombinant virus has potential to be developed as a pan-lyssavirus vaccine.

## 1. Introduction

The *Lyssavirus* genus consists of 14 viral species and three putative species, grouped into three phylogroups [1,2,3]. While all members of the lyssavirus genus are capable of causing a fatal encephalitic disease, rabies virus (RABV) in phylogroup I is responsible for most human and animal rabies cases and is globally distributed. Other lyssavirus species have a more restricted distribution, with Lagos bat lyssavirus (LBV), Mokola lyssavirus (MOKV), Duvenhage lyssavirus (DUVV), Shimoni bat lyssavirus (SHIBV) and Ikoma lyssavirus (IKOV) exclusively identified from Africa. LBV is associated with Pteropodidae bat species [4,5,6] and although no human infections have been reported to date, fatal spill-over into dogs [7,8], cats [5] and a mongoose [9] have been reported. Although case reports of LBV are limited, pathogenicity studies in mice have indicated that the virus is pathogenic to mice when inoculated via the intramuscular (i.m.) and intracranial (i.c.) route with distinct pathogenicity profiles observed between different LBV lineages [6,10,11].

The lyssavirus genome codes for five proteins; the nucleo- (N), phospho- (P), matrix- (M), glyco (G) and RNA polymerase protein (L) and their cooperative effect in pathogenesis have been shown [12,13,14,15]. Gene exchange between RABV strains with different pathogenicity profiles has been performed in previous studies mostly focusing on the G protein [13,16,17,18,19,20]. Different pathogenic mechanisms have been linked to this protein, including interaction with the cell surface molecules, p75 neurotropin receptor [21,22], nicotinic acetylcholine receptor [23] and neural cell adhesion molecule [24], to facilitate binding and entry into the cell. Controlled expression levels of the G gene are important to prevent apoptosis of neuronal cells and they allow transport of the virus to the central nervous system [25]. A number of studies have demonstrated that replacement of the G gene from a non-pathogenic RABV strain with that of a pathogenic strain results in a pathogenic RABV strain [13,16,17,20].

Replacement of the M gene of Ni-CE strain (non-pathogenic both i.c. and i.m.) with that of the Nishigahara strain (pathogenic both i.c. and i.m.) was shown to result in a pathogenic strain when mice were inoculated i.c. [15]. Pulmanausahakul et al. (2008) showed that replacement of both the M and G gene of a non-pathogenic strain with that of a pathogenic strain resulted in increased pathogenicity when compared with replacing only the G gene. Substitution of the M gene alone did not result in a pathogenic strain. In another study, replacement of the M, G, G-L intergenic region and L gene from a pathogenic strain with that of a non-pathogenic strain resulted in a non-pathogenic strain when mice were inoculated i.m., but when only the G, G-L region and L gene were replaced, the virus was pathogenic [12]. The M gene of LBV and MOKV has also been shown to induce apoptosis in vitro in neuroblastoma and HeLa cells [26]. Apoptosis has been reported to be inversely proportional to pathogenicity of RABV [25,27].

The recombinant RABV backbones (non-pathogenic i.m.) were used for interspecies gene or partial gene replacement between RABV and other lyssaviruses in previous studies. Interspecies G protein substitution was performed between a RABV vaccine strain (SAD B19) and European bat lyssavirus type 1 and 2 (EBLV-1 and EBLV-2) [18]. The RABV with an EBLV-1 G protein was shown to cause higher mortality when inoculated i.m. compared to inoculation with a RABV recombinant backbone. Genz et al. (2012) also generated chimeric RABV and EBLV-1 or EBLV-2 G protein in a backbone of RABV (SAD B19). The chimeric G protein consisted of the cytoplasmic tail from RABV while the transmembrane and ectodomain was from the EBLVs. The recombinant viruses had comparable growth kinetics in vitro and were lethal to mice when inoculated i.c. [28]. Interspecies M protein substitution between the RABV vaccine strain (SAD B19) and EBLVs (EBLV-1 and EBLV-2) were performed and it was shown that the M protein plays a role in intracellular virus accumulation [29]. Although gene exchange between different strains of RABV has been done, no study has performed gene exchange between RABV and LBV [12].

All currently licensed lyssavirus vaccines are based on a RABV backbone. These vaccines have been shown to protect against members of phylogroup I lyssaviruses [30,31,32,33,34,35]; however, there appears to be no vaccine affording protection against phylogroup II and III lyssaviruses [2,30,36,37,38]. Mice vaccinated with RABV vaccine produced significantly lower virus-neutralizing antibodies (VNAs) against DUVV when compared to RABV VNAs.

The generation of recombinant viruses using reverse genetics systems has led to the construction of safe and immunogenic rabies vaccines. Recombinants included the introduction of two or three G genes [39,40,41,42], mutation of pathogenic domains on the G gene [39,40], deletion of genes required for replication [43] and introduction of inflammatory cytokines [44,45], chemokines [46] and pro-apoptotic [47] genes into the RABV genome. Recombinant RABV vaccines containing two RABV G genes were shown to express high levels of the G protein, and were more immunogenic than parental strains [39,41,42]. To increase the safety of the vaccine, mutations of Asn 194 to Ser and Arg 333 to Glu were introduced in the G gene [39,48,49]. A recombinant RABV vaccine with three G genes was investigated [40] and the triple G vaccine was shown to be non-pathogenic to juvenile mice, adult mice deficient in some immune functions, and normal adult mice when inoculated via the i.c. route. The recombinant vaccine protected mice against lethal challenge with RABV and also protected mice when administered as post-exposure prophylaxis.

The aim of this study was to determine the importance of the LBV M and G genes in pathogenesis using a recombinant RABV and replacing the G gene, as well as both the M and G gene, with that of LBV. In addition, the pathogenicity and immunogenicity of a RABV backbone with a LBV G gene sandwiched between two RABV G genes was determined. We demonstrate that the RABV backbone can express functional LBV genes, and that both the M and G genes of LBV play a role in pathogenicity. The feasibility of generating a pan-African lyssavirus vaccine that can induce neutralizing antibodies against at least phylogroup I and II lyssaviruses was demonstrated.

## 2. Material and Methods

### 2.1. Virus Isolates, Recombinant Viruses and Cell Lines

The recombinant viruses, SPBN and SPBNGAS-GAS-GAS, were constructed in previous studies [40,50]. The LBVAFR1999 [51,52] was maintained in mouse neuroblastoma (MNA) cells (C-1300). MNA and BSR-T7 (a clone of BHK-21) [53] cells were grown in an atmosphere of 37 °C and 5% CO_2_ using Dulbecco’s modified Eagle’s medium (DMEM/F12) (Lonza, Verviers, Belgium) supplemented with 10% fetal calf serum (Lonza, Verviers, Belgium) and 1% antibiotics (penicillin [100 units/mL], streptomycin [100 μg/mL] and amphotericin B [0.25 μg/mL]) (Lonza, Verviers, Belgium).

### 2.2. Construction of the Recombinant Viruses

**Figure 1:**
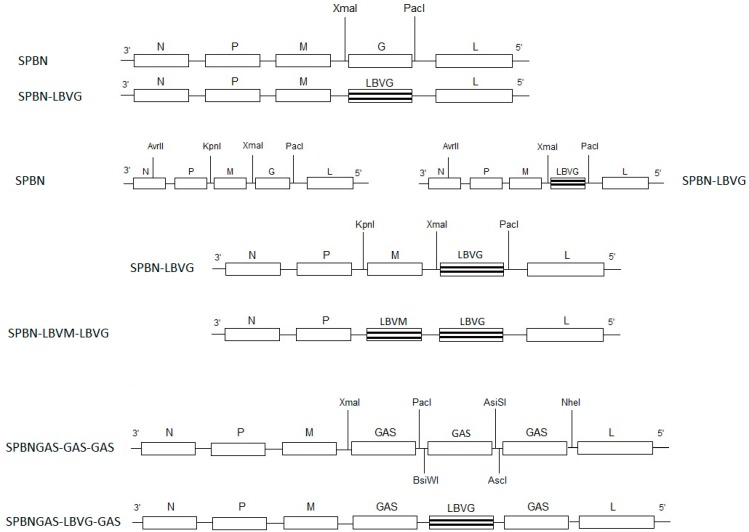
Schematic representation of the construction of recombinant viruses. Restriction enzyme sites are indicated (*XmaI*, *PacI*, *AvrII*, *KpnI*. *BsiWI*, *AsiSI*, *NheI* and *AscI*). GAS represents the SPBN G gene with two amino acid substitutions (Asn 194 to Ser and Arg 333 to Glu). The following abbreviations were used: N, nucleoprotein; M, matrix protein; G, glycoprotein; L, RNA-dependent RNA polymerase. LBVM and LBVG represent the LBV M and G gene respectively.

Total RNA was isolated from LBVAFR1999 cell culture material using TRIzol^®^ reagent (Invitrogen, Carlsbad, CA, USA) according to manufacturer’s instructions. To replace the G gene of SPBN with that of the LBVAFR1999, the G gene was amplified from the LBVAFR1999 RNA using *Pfu* DNA polymerase (Promega, Madison, WI, USA) and the primers LBVXmaI and LBVPacI containing restriction enzyme sites *XmaI* and *PacI* respectively (Table 1). The amplified PCR product was digested with *XmaI* and *PacI* (New England Biolabs, Ipswich, MA, USA) and then ligated to pSPBN previously digested with *XmaI* and *PacI*. The resulting plasmid was designated pSPBN-LBVG (Figure 1). To replace both the M and G genes of SPBN with those of the LBVAFR1999, the *KpnI* site was introduced upstream of the pSPBN-LBVG M gene start signal, through digestion of another pSPBN (that contains the *KpnI* site) with *AvrII* and *KpnI* (New England Biolabs, Ipswich, MA, USA) (Figure 1). The digested fragment was ligated to pSPBN-LBVG previously digested with the same restriction enzymes (*AvrII* and *KpnI*). LBVAFR1999 M gene was synthesized (GenScript, Piscataway, NJ, USA) to contain the *KpnI* and *XmaI* restriction enzyme sites. The M gene was digested with the *KpnI* and *XmaI* and then ligated to pSPBN-LBVG previously digested with *KpnI* and *XmaI*. The resulting plasmid was designated pSPBN-LBVM-LBVG (Figure 1). To construct the recombinant virus with a LBVAFR1999 G gene sandwiched between two RABV G genes, the G gene was amplified from the LBVAFR1999 RNA using *Pfu* DNA polymerase (Promega, Madison, WI, USA) and the primers LBVEcoRIBsiWI and LBVXbaIAsiSI (Table 1). The amplified PCR product was digested with *BsiWI* and *AsiSI* (New England Biolabs, Ipswich, MA, USA) and then ligated to pSPBNGAS-GAS-GAS previously digested with *BsiWI* and *AsiSI*. The resulting plasmid was designated pSPBNGAS-LBVG-GAS (Figure 1).

### 2.3. Rescue of the Recombinant Viruses

The recombinant viruses were rescued as described previously [40,50]. Briefly, 250 μL of FuGENE (Promega, Madison, WI, USA) in serum-free DMEM (Lonza, Verviers, Belgium) was used to transfect BSR-T7 cells with 10 μg of full length plasmids (pSPBN-LBVG, pSPBN-LBVM-LBVG or pSPBNGAS-LBVG-GAS), helper plasmids (5 μg of pTIT-N, 2.5 μg of pTIT-P, 2.5 μg of pTIT-L, 1 μg of pTIT-G) and 2 μg of pTIT-T7 (a plasmid expressing the T7 RNA polymerase). Supernatants were transferred onto MNA cells in 12-well plates (Greiner Bio-one, Frickenhausen, Germany) 72 h post-transfection and incubated in an atmosphere of 37 °C and 5% CO_2_ for 72 h. Positive cells were determined by immunostaining [54] using fluorescein isothiocyanate conjugate (FITC)-labeled RABV N gene specific antibody (Centocor Inc., Malvern, PA, USA). The nucleotide sequence of the inserted genes was confirmed by sequencing as described previously [10].

### 2.4. Amplification and Titration of the Rescued Recombinant Viruses

MNA cells in 6-well plates (Greiner Bio-one, Germany) were infected with supernatant from wells that were positive during transfection and incubated in an atmosphere of 37°C and 5% CO_2_ for 72 h. The supernatant was harvested and then immunostaining was performed on the plates using FITC-labeled RABV N gene specific antibody (Centocor Inc.). Supernatant from positive wells in the 6-well plates (Greiner Bio-one, Germany) was subsequently amplified in MNA cells in T75 flasks (Corning Incorporated, Corning, NY, USA) and incubated in an atmosphere of 37 °C and 5% CO_2_ for 72 h to prepare virus stocks. To determine virus yield, MNA cells in 96-well plates (Greiner Bio-one, Frickenhausen, Germany) were infected with ten-fold serial dilutions of the virus stocks and incubated in an atmosphere of 37 °C and 5% CO_2_ for 48 h. All titrations were performed in triplicate. Immunostaining using FITC-labeled RABV N gene-specific antibody (Centocor, Inc.) was performed on the plates and the 50% tissue culture infectious dose (TCID_50_) was determined according to the method described elsewhere [55].

### 2.5. Lagos Bat Virus Glycoprotein Expression by the Recombinant Viruses

Neutralization of the SPBN-LBVG, SPBN-LBVM-LBVG, SPBNGAS-LBVG-GAS, SPBN (as a negative control) and LBVAFR1999 (positive control) was determined by the rapid fluorescent focus inhibition test (RFFIT) [6,56] using LBV antiserum. This serum was previously collected from bats in South Africa [57] and was found to have virus-neutralizing antibodies (VNAs) against LBV but not against RABV. Four different dilutions (1:10, 1:100, 1:400 and 1:1000) of the serum were used for the neutralization assay. The titer of the challenge viruses used in the neutralization assay was 1 × 10^3^ FFU/mL. The virus neutralization index (VNI) was calculated by subtracting the virus titer of the untreated challenge virus with the virus titer of the LBV antiserum- treated challenge virus.

### 2.6. Single- and Multi-Step Growth Assays

Single- and multi-step growth assays were performed to determine the growth pattern of the different recombinant viruses. MNA cells in T25 culture flasks were infected with virus inoculums at a multiplicity of infection (MOI) of 2 or 0.01 for the single- and multi-step growth curves respectively, and incubated for 2 h in an atmosphere of 37 °C and 5% CO_2_. The virus inoculum was removed, the cells were washed three times with 5 mL of PBS (Lonza, Verviers, Belgium) and then 7 mL of RPMI (containing 0.2% bovine serum albumin (BSA)) (Lonza) was added to the flask. At 24, 48, 72 and 96 h post infection, 100 μL of the supernatant was collected and virus titration was performed as described above. The virus titer was expressed as focus-forming units per mL (FFU/mL).

### 2.7. Experimental Infections of Mice

Six-week-old mice (Crl:CD1 [ICR]) (Onderstepoort Biological Products, Pretoria, South Africa) were used for experimental infection. The experiments were performed in a biosafety level 3 (BSL-3) laboratory at the Department of Medical Virology, Faculty of Health Sciences, University of Pretoria, South Africa. Ethical approval (EC052-13) for these experiments was granted by the University of Pretoria Animal Ethics Committee and Section 20 approval according to the Animal Disease Act, 1984, was granted by the Department of Agriculture, Forestry and Fishery, South Africa. Mice were housed in a group of five in HEPA-filtered OptiMICE cage units (Animal Care Systems, Centennial, CO, USA). For the pathogenesis study, groups of 4 to 5 mice were inoculated via the i.c. or i.m. (in the hind thigh) route with 5 × 10^6^ TCID_50_/50 μL of virus inoculums (LBVAFR1999, SPBN, SPBN-LBVG, SPBN-LBVM-LBVG, SPBNGAS-LBVG-GAS or SPBNGAS-GAS-GAS) using sterile 1 mL syringes (Becton Dickinson, Franklin Lakes, NJ, USA). For the vaccine study, 10 mice were vaccinated on day 0 and boosted on day 20 with SPBNGAS-LBVG-GAS via the i.m. (in the hind thigh) route with 1 × 10^5^ TCID_50_/50 μL of virus inoculum using sterile 1 ml syringes (Becton Dickinson). Mice were monitored daily for 43 days for the pathogenesis study and 30 days for the vaccine study. Death and clinical signs such as not eating, paralysis, confusion, running in circles, weight loss, ruffled fur and restlessness were recorded daily. The mice were euthanized by i.m. inoculation with a mixture of ketamine (Anaket-V) (35 mg/kg body mass) and xylazine (Chanazine or Rompun) (5 mg/kg body mass) upon development of clinical signs or at the end of the experiment in cases where clinical signs were not observed. Brains were collected and analyzed for the presence of lyssavirus antigen using anti-rabies polyclonal FITC conjugate (Rabies Unit, Onderstepoort Veterinary Institute, Agricultural Research Council, Pretoria, South Africa) in the fluorescent antibody tests (FAT) as described by Dean et al. 1996. Blood was collected in BD Microcontainer^TM^ (Becton Dickinson) from the saphenous vein using 80 μL capillaries (Lasec, Cape Town, South Africa) on days 13 and 28 after primary vaccination. Mice were anesthetized with isoflurane (Piramal Heathcare, India) prior to bleeding. The blood was centrifuged at 13,400 rpm for 5 min, and serum collected in 2 mL microcentrifuge tubes (Quality Scientific Plastics, San Diego, CA, USA) and stored at −20 °C until use.

### 2.8. Rapid Fluorescent Focus Inhibition Test (RFFIT)

Virus-neutralizing antibodies (VNAs) from serum of mice vaccinated with SPBNGAS-LBVG-GAS were determined by a modification of the rapid fluorescent focus inhibition test (RFFIT) [6,56]. The following challenge viruses were used: RABV (challenge virus standard [CVS]), LBV (LBVAFR1999), MOKV (252/97) and DUVV (DUVVSA2006). Eight different dilutions were tested: 1:10, 1:25, 1:65, 1:160, 1:400, 1:1000, 1:2500 and 1:6100. The VNA titer was indicated as the highest dilution where there was a 50% reduction in the number of foci. Since there are no reference sera available for LBV, MOKV and DUVV, the VNA titer for RABV were not converted to international units.

### 2.9. Statistical Analyses

For the pathogenesis study, Fisher’s exact test (CI = 95%) was used to determine the difference in survival between the different groups of mice. For the single- and multi-step growth curves, as well as the vaccine study, statistical analysis was performed using SPSS version 20, licensed to the University of Pretoria. One-way ANOVA was performed followed by Tukey HSD as a post-hoc test.

## 3. Results

### 3.1. Confirmation of LBV G Protein Expression by Recombinant Viruses

A virus neutralization test (RFFIT) using LBV antiserum was employed to ascertain that the LBV G protein is expressed. The neutralization indices tests shown in Table 2 reveal that SPBN-LBVG, SPBN-LBVM-LBVG, SPBNGAS-LBVG-GAS and LBVAFR1999 (positive control) but not SPBN (negative control) were completely neutralized by the LBV anti-serum.

### 3.2. In Vitro Growth of Recombinant Viruses in MNA Cells

**Figure 2:**
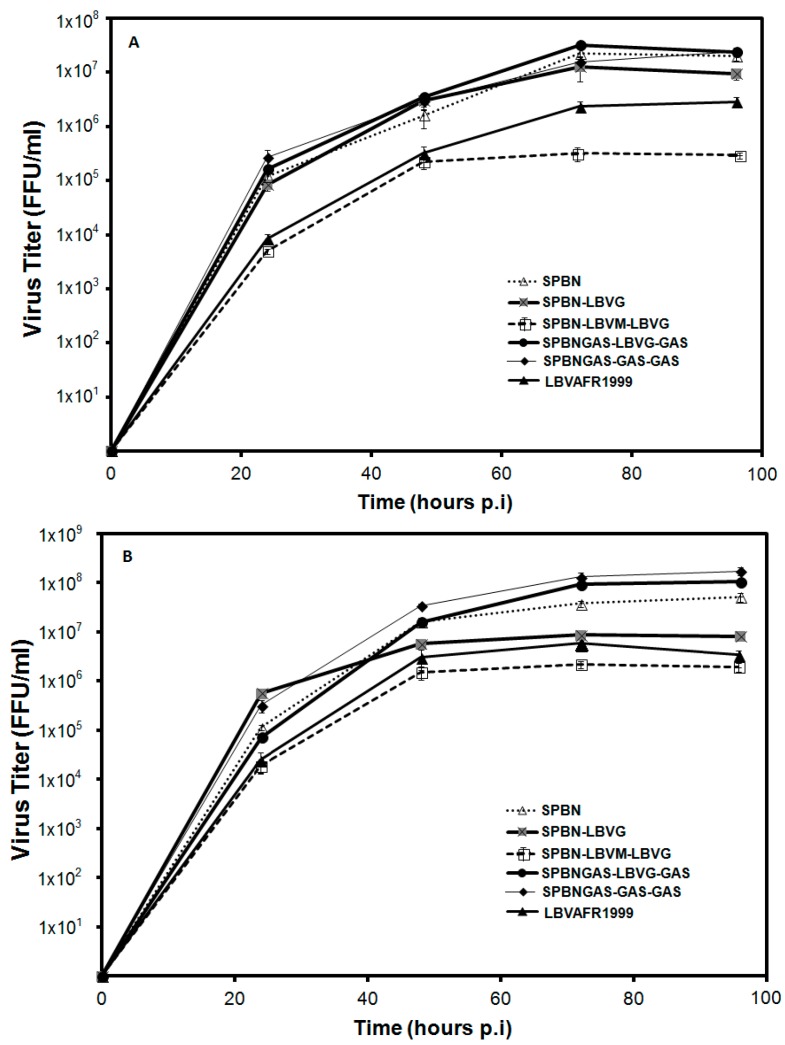
Multi-step (**A**) and single-step (**B**) growth curves of recombinant rabies viruses in MNA cells. MNA cells were infected with the recombinant viruses (SPBN, SPBN-LBVG, SPBN-LBVM-LBVG, SPBNGAS-GAS-GAS or SPBNGAS-LBVG-GAS) or LBVAFR1999 at a MOI of 0.01 (**A**) or a MOI of 2 (**B**) and incubated at 37 °C. At 24, 48, 72 and 96 h post infection, the recombinant viruses were harvested and titration was performed. Data represent the mean of triplicate experiments.

Viral replication rate has been linked to pathogenicity of the virus [12,13]. We investigated the growth kinetics of recombinant RABVs in which the G only or M and G genes were replaced with corresponding LBV genes (Figure 2). Multi-step growth curves (Figure 2A) showed that SPBN and SPBNGAS-GAS-GAS had the highest growth rate followed by SPBN-LBVG and SPBNGAS-LBVG-GAS. While there was no statistically-significant difference between the growth rate of these viruses (*p* > 0.05), the growth rate of SPBN-LBVM-LBVG was lower than that of the other viruses including LBVAFR1999. The single-step growth curves yielded similar results, with SPBNGAS-GAS-GAS, SPBNGAS-LBVG-GAS and SPBN producing the highest titers, and SPBN-LBVM-LBVG and LBVAFR1999 again producing the lowest titers (Figure 2B). SPBN-LBVG also produced a lower titer, however it was still higher than that of SPBN-LBVM-LBVG.

### 3.3. Pathogenicity of Recombinant Viruses in Mice

**Figure 3:**
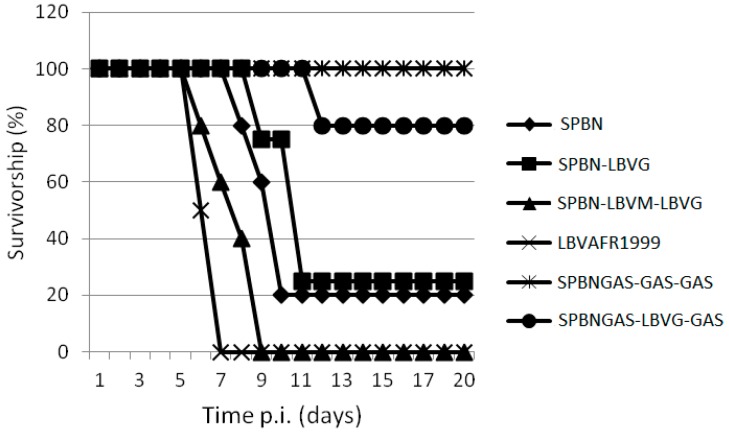
Pathogenicity of SPBN, SPBN-LBVG, SPBN-LBVM-LBVG, LBVAFR1999, SPBNGAS-GAS-GAS and SPBNGAS-LBVG-GAS in mice. Groups of (Crl:CD1 [ICR]) mice were inoculated via the i.c. route with 5 × 10^6^ TCID_50_/50 μL of virus. The groups consisted of 5 mice except for SPBN-LBVG and LBVAFR1999 which consisted of 4 mice per group. The experiment was terminated after 43 days, but no clinical signs were observed 12 days post-infection.

**Figure 4:**
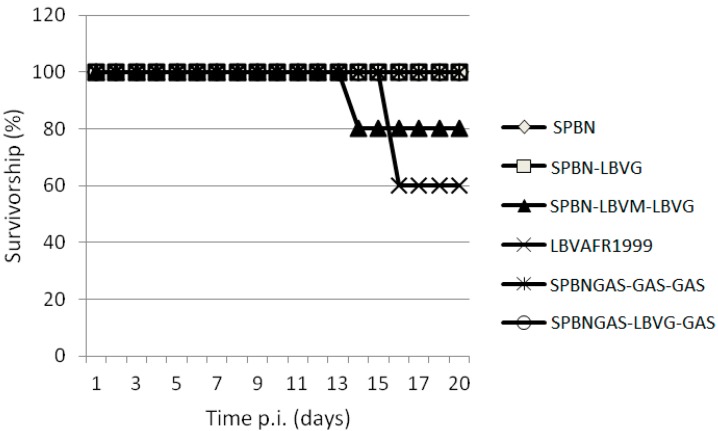
Pathogenicity of SPBN, SPBN-LBVG, SPBN-LBVM-LBVG, LBVAFR1999, SPBNGAS-GAS-GAS and SPBNGAS-LBVG-GAS in mice. Groups of 5 (Crl:CD1 [ICR]) mice were inoculated via the i.m. route with 5 × 10^6^ TCID_50_/50 μL of virus. The experiment was terminated after 43 days, but no clinical signs were observed 16 days post infection.

The ability of various recombinant viruses to cause lethal encephalitis in mice was compared. Virus infection of the brain was confirmed postmortem by FAT. All viruses, except SPBNGAS-GAS-GAS, caused mortality in mice after i.c. inoculation and all the mice that died exhibited signs of rabies including walking in circles, hind leg paralysis, weight loss and ruffled fur. The mortality rate ranged from 0% to 100% (Figure 3 and Table 3). Mice inoculated with LBVAFR1999 and SPBN-LBVM-LBVG had the highest mortality rate (100%) while those inoculated with SPBNGAS-LBVG-GAS had the lowest mortality rate (20%). After i.m. inoculation of recombinant viruses, only LBVAFR1999 and SPBN-LBVM-LBVG caused mortality (Figure 4). The rates were 40% for LBVAFR1999 and 20% for SPBN-LBVM-LBVG with incubation periods of 16 and 14 days, respectively. These results show that both the M and G proteins of LBV play a role in the pathogenesis of LBV.

### 3.4. VNA Response of Mice Vaccinated with SPBNGAS-LBVG-GAS

For primary vaccination (Table 4), VNA titers against LBV and MOKV were significantly lower (*p* < 0.05) compared to RABV (with the exception of VNA titer for RABV and LBV at day 28, where there were no significant differences). There was no significant difference (*p* > 0.05) in VNA titer between RABV and DUVV (Table 4). Booster vaccination resulted in significantly higher (*p* < 0.05) VNA titers against all challenge viruses used. This VNA analysis demonstrates that SPBNGAS-LBVG-GAS can induce cross-reactive antibodies capable of neutralizing different lyssaviruses.

## 4. Discussion

The successful rescue of RABV recombinant viruses expressing LBV proteins, indicates that RABV genes can interact with the LBV M and G genes, similar to previous studies [18,28,29,58]. Finke et al. (2010) and Marston et al. (2013) showed that EBLV-1 and EBLV-2 genes can interact with RABV genes by respectively performing replacement of the M and G genes from RABV with those from EBLV-1 or EBLV-2. In addition to these studies, Genz et al. (2012) replaced the transmembrane and ectodomain of RABV G protein with those of EBLV-1 or EBLV-2. Neutralization of SPBN-LBVG, SPBN-LBVM-LBVG and SPBNGAS-LBVG-GAS with LBV antiserum confirmed that the LBV G protein is expressed by these recombinant viruses. Failure of the same serum to neutralize SPBN shows that the VNAs were directed against the LBV G protein [59,60]. A G gene-deleted RABV (SAD ΔG) was previously shown to be unable to spread in vivo and in vitro, and also failed to produce infectious virus particles when passaged in cells that were not transfected with the plasmid expressing the G protein [61,62]. The SAD ΔG was also shown to be non-pathogenic when inoculated i.c. [62] and in addition, the M gene-deleted RABV had a reduced viral titer by about 500 000-fold [63]. The spread of SPBN-LBVG and SPBN-LBVM-LBVG in cells that were not transfected with the relevant exchanged genes also indicated that the recombinant viruses functionally expressed these genes. Western blotting could not be used to determine LBV M and G genes expression because of the lack of suitable antibodies directed against these proteins.

All the viruses in this study, except for SPBNGAS-GAS-GAS, were pathogenic to mice after i.c. inoculation (Table 3), and LBVAFR1999 and SPBN-LBVM-LBVG infection resulted in 100% mortality. The 20% mortality rate observed with SPBNGAS-LBVG-GAS compared to 0% mortality of SPBNGAS-GAS-GAS indicated that the non-pathogenic RABV G (GAS) genes were not dominant over the pathogenic phenotype of the LBV G gene. Previously, it was shown that i.c. inoculation of mice with a double-G gene recombinant virus (SPBNGAK-GAK) resulted in 70% mortality [48]. However, when one of the G genes was made non-pathogenic (by mutation of Lys 194 to Asn), the recombinant virus only caused 10% mortality to mice, indicating dominance of the non-pathogenic G over the pathogenic G gene [48]. This indicates that in addition to multiple G genes that result in over-expression and therefore reduced pathogenicity [25], the pathogenic domains of the G genes must be eliminated. Inoculation of the viruses into mice i.m. showed that only LBVAFR1999 and SPBN-LBVM-LBVG were pathogenic. In contrast, the SPBN-LBVG was not pathogenic despite expressing the G gene from a pathogenic LBV. It was previously shown that replacement of a non-pathogenic RABV G gene with that of a pathogenic RABV results in a pathogenic RABV, but with reduced pathogenicity compared to the parental strain [13,16,18]. In other studies, replacement of a non-pathogenic RABV G gene with a pathogenic RABV did not result in a pathogenic strain [16,64]. Replacement of a G gene from a non-pathogenic RABV (SN-10) with the G gene from a number of street RABV variants (pathogenic i.m.) resulted in a non-pathogenic RABV strain when inoculated i.m. to mice [16,64], as also confirmed in our study where the SPBN-LBVG was non-pathogenic after i.m inoculation. Replacement of both the M and G gene of a non-pathogenic RABV with that of a pathogenic RABV was shown to result in increased pathogenicity as compared to the replacement of only the G gene, indicating the importance of the M gene in pathogenicity [12,13]. In our study, the SPBN-LBVM-LBVG was pathogenic i.m. while the SPBN-LBVG was non-pathogenic, which is in agreement with the previous studies indicating the role of the M gene in pathogenicity [12,13,15].

Differences in recombinant virus growth was determined in MNA cells by multi-step and single-step growth curves. SPBN and SPBN-LBVG showed the same growth rate in the multi-step growth curve, indicating that the LBV G gene is able to interact optimally with the RABV M gene during encapsidation and budding. Notably, LBVAFR1999 and SPBN-LBVM-LBVG produced the lowest viral titers during single- and multi-step growth kinetics, which is in agreement with previous findings that pathogenic RABV strains have lower replication rates than non-pathogenic strains [12,13,48]. This, thought to be one of the mechanisms by which lyssaviruses evade the host defense mechanisms, is consistent with our finding that only the LBVAFR1999 and SPBN-LBVM-LBVG were pathogenic when inoculated i.m. in mice. The single-step growth curve indicated no significant (*p* > 0.05) difference between SPBN, SPBNGAS-LBVG-GAS and SPBNGAS-GAS-GAS. These viruses maintained higher titers in both the single- and multi-step growth curves, indicating that insertion of multiple G genes and the LBVAFR1999 G gene did not negatively influence the growth of SPBNGAS-LBVG-GAS and SPBNGAS-GAS-GAS.

By replacing the M and G genes between laboratory-adapted SPBN and the wild-type LBVAFR1999 isolate, we have attempted to address the importance of the M and G genes in the pathogenicity of LBV. Our results emphasize again that the G protein is important in pathogenicity of lyssaviruses, and that there is a cooperative effect of other lyssavirus genes as shown by increased pathogenicity when both the SPBN M and G genes were replaced by those of LBV.

The SPBNGAS-LBVG-GAS produced high levels of VNAs against RABV and DUVV 13 days post-primary vaccination in mice and the level was comparable to that produced by vaccination with SPBNGAS-GAS-GAS (data not shown). In previous studies, SPBNGAS-GAS-GAS produced high levels of RABV VNA and protected mice against lethal challenge with RABV, therefore suggesting that SPBNGAS-LBVG-GAS will offer the same level of protection [39,40]. In addition, SPBNGAS-LBVG-GAS also produced high levels of VNAs capable of cross-neutralizing DUVV. Although serum from RABV-vaccinated mice has already been shown to cross-neutralize DUVV [30] the VNA titers against DUVV were significantly lower than those against RABV. In our study, the DUVV VNA titers induced by SPBNGAS-LBVG-GAS were not significantly different from the RABV VNA titers (*p* > 0.05). Previous studies on cross-reactivity of RABV vaccines to other phylogroup I lyssaviruses indicated that a vaccine derived from the Pasteur virus (PV) strain protected mice from EBLV-1 challenge, while vaccines derived from Pitman-Moore (PM) and LEP-Flury (LEP) strains failed to protect mice against EBLV-1 infection [31]. However, in another study, a vaccine derived from the PM strain was shown to produce VNA that neutralize EBLV-1 and protect mice against lethal challenge [35]. This indicates that different strains from the same lyssavirus species can result in different protection levels.

Mice vaccinated with SPBNGAS-LBVG-GAS also produced VNAs against LBV and MOKV although the titers were significantly lower (*p* < 0.05) compared to RABV, with the exception of VNA titer of RABV and LBV at day 28, where no significant difference was observed. MOKV VNA titers were significantly lower (*p* < 0.05) than LBV VNA titers on day 13. Previous studies did report cross-neutralization between MOKV and LBV [30,65,66]; however, the titer was always higher when the G protein of the challenge virus was identical to the G protein of vaccine. Cross-neutralization against SHIBV was not tested in this study but it is likely that cross-neutralization will occur with other phylogroup II viruses when SPBNGAS-LBVG-GAS is used as vaccine [38]. Booster vaccination resulted in significantly higher (*p* < 0.05) VNA titer against all challenge viruses used. Mice were not challenged in this study; however, the high VNA titer produced by SPBNGAS-LBVG-GAS especially after booster vaccination indicates that this recombinant vaccine will likely protect mice against lethal challenge with RABV, LBV, MOKV and DUVV. VNA titer is regarded to be an important factor in protecting against virus challenge and can be correlated with survival, but this will have to be confirmed in challenge studies in mice in the future [67].

In future studies, the expression of LBV G should be increased by changing the gene order of the LBV G gene or by insertion of an additional G gene to determine if the level of LBV and MOKV VNA increases without booster vaccination. Although VNA titers against LBV and MOKV were lower compared to RABV and DUVV during primary vaccination, the significant increase in VNA titer after booster vaccination warrants further investigation of the efficacy of SPBNGAS-LBVG-GAS to protect against phylogroup II lyssaviruses.

## Figures and Tables

**Table 1:** Primers used for amplification or sequencing of the Lagos bat virus matrix and glycoprotein genes.

Primer	Sequence in 5′ to 3′ Direction *	Gene Targeted
LBVXmaI	TATCCCCCCGGGAAG*ATG*AGTCAATTGTTCTCAACC	G gene
LBVPacI (to be used with LBVXmaI)	CCGACCTTAATTAAGG*TTA*GACACTTGATGTCTCTTTATATG	G gene
LBVEcoRIBsiWI	GCAGAATTCCGTACGAAG*ATG*AGTCAATTGTTCTCAACCTTCAT	G gene
LBVXbaIAsiSI (to be used with LBVEcoRIBsiWI)	TAATCTAGAGCGATCGCCGT*TTA*GACACTTGATGTCTCTTTATATGA	G gene
LBV1319F (binds on position 1319 on the open reading frame of the LBVAFR1999 G gene, EF547432)	CGACTTCGTCGATGTCCACATGCC	G gene
LBV255R (binds on position 255 on the open reading frame of the LBVAFR1999 G gene, EF547432)	GTTGGTGTATGTGACTGCCTCG	G gene
RabC2121F (to be used with LBV255R [binds on position 2121 on the complete genome of RABV, NC_001542])	CAGTGGAGGCTGAGATCGCTC	M gene

* Restriction enzyme sites are indicated in bold or underlined, start/stop codons are in italics.

**Table 2:** Neutralization of recombinant viruses with the different dilutions of the anti-Lagos bat virus serum as determined by the rapid fluorescent focus inhibition test (RFFIT).

Challenge Virus *	Virus Neutralization Index (VNI) *^#^* Anti-LBV Serum Dilution			
	1:10	1:100	1:400	1:1000
LBV (LBVAFR1999) (positive control)	10^3^	10^3^	10^3^	10^0^
SPBN (negative control)	10^0^	10^0^	10^0^	10^0^
SPBN-LBVG	10^3^	10^3^	10^3^	10^0^
SPBN-LBVM-LBVG	10^3^	10^3^	10^3^	10^0^
SPBNGAS-LBVG-GAS	10^3^	10^3^	10^0^	10^0^

* 10^3^ FFU of each challenge virus was used; *^#^* VNI = virus titer of untreated challenge virus – virus titer of challenge virus incubated with anti-LBV serum.

**Table 3:** Intracranial experimental infections of mice with recombinant viruses.

Virus	Mean Incubation Period, s.d. and Range of Incubation Periods (Days)		Number of Mice that Died per Group of Mice Inoculated i.c.
	Mean and s.d.	Range	
LBVAFR1999	6.5 ± 0.58	6–7	4/4 (100%)
SPBN	9.25 ± 0.96	8–10	4/5 (80%)
SPBN-LBVG	10.33 ± 1.15	9–11	3/4 (75%)
SPBN-LBVM-LBVG	7.8 ± 1.30	6–9	5/5 (100%)
SPBNGAS-GAS-GAS	-	-	0/5 (0%)
SPBNGAS-LBVG-GAS	12 ± NA	12	1/5 (20%)

N/A = no s.d. since only one mouse died; s.d. = standard deviation; i.c. = intracranial.

**Table 4:** Virus neutralizing antibodies in mice vaccinated with SPBNGAS-LBVG-GAS as determined by the rapid fluorescent focus inhibition antibody test (RFFIT).

Mouse No.	Neutralization Dilution * (RABV-CVS) ^#^		Neutralization Dilution * (DUVVSA2006) ^#^		Neutralization Dilution * (LBVAFR1999) ^#^		Neutralization Dilution * (MOKV252/97) ^#^	
	Day 13	Day 28	Day 13	Day 28	Day 13	Day 28	Day 13	Day 28
Mouse 1	1:400	1:2500	1:160	1:1000	1:160	1:1000	1:65	1:400
Mouse 2	1:160	1:2500	1:65	1:400	1:65	1:400	1:10	1:160
Mouse 3	1:1000	1:6100	1:400	1:2500	1:400	1:6100	1:160	1:1000
Mouse 4	1:400	1:1000	1:400	1:1000	1:160	1:400	1:65	1:400
Mouse 5	1:400	1:2500	1:160	1:1000	1:65	1:400	1:25	1:400
Mouse 6	1:400	1:1000	1:160	1:1000	1:160	1:1000	1:65	1:400
Mouse 7	1:160	1:1000	1:65	1:1000	1:65	1:400	1:25	1:400
Mouse 8	1:400	1:2500	1:400	1:2500	1:160	1:1000	1:65	1:400
Mouse 9	1:400	1:2500	1:400	1:1000	1:160	1:1000	1:160	1:1000
Mouse 10	1:1000	1:6100	1:400	1:6100	1:400	1:2500	1:160	1:1000
**Range** ^~^	1:160–1:1000	1:1000–6100	1:65–1:400	1:400–1:6100	1:65–1:400	1:400–1:6100	1:10–1:160	1:400–1:1000

Key: * Represents the dilution where there was 50% virus neutralization by the serum. The experiments were performed in triplicate; *^#^* Indicates the challenge virus used; ^~^ Indicates the range of virus neutralization titer between the different mice.

## References

[1] Dietzgen R.G., Calisher C.H., Kurath G., Kuzmin I.V., Rodriguez L.L., Stone D.M., Tesh R.B., Tordo N., Walker P.J., Wetzel T., King A.M.Q., Adams M.J., Carstens E.B., Lefkowitz E.J. (2011). Family Rhabdoviridae. Virus Taxonomy: Ninth Report of the International Committee on Taxonomy of Viruses.

[2] Banyard A.C., Fooks A.R. (2017). The impact of novel lyssavirus discovery. Mcrobiol. Aust..

[3] ICTV. http://ictvonline.org/virusTaxonomy.asp/.

[4] Markotter W., Kuzmin I., Rupprecht C., Nel L. (2008). Phylogeny of Lagos bat virus: Challenges for lyssavirus taxonomy. Virus Res..

[5] Swanepoel R., Coetzer J.A.W., Tustin R.C. (2004). Rabies. Infectious Diseases of Livestock: With Special Reference to Southern Africa.

[6] Kuzmin I.V., Niezgoda M., Franka R., Agwanda B., Markotter W., Beagley J.C., Urazova O.Y., Breiman R.F., Rupprecht C.E. (2008). Lagos bat virus in Kenya. J. Clin. Microbiol..

[7] Mebatsion T., Cox J.H., Frost J.W. (1992). Isolation and characterization of 115 street rabies virus isolates from Ethiopia by using monoclonal antibodies: Identification of 2 isolates as Mokola and Lagos bat viruses. J. Infect. Dis..

[8] Markotter W., Van Eeden C., Kuzmin I., Rupprecht C., Paweska J., Swanepoel R., Fooks A., Sabeta C., Cliquet F., Nel L. (2008). Epidemiology and pathogenicity of African bat lyssaviruses. Dev. Biol..

[9] Markotter W., Kuzmin I., Rupprecht C.E., Randles J., Sabeta C.T., Wandeler A.I., Nel L.H. (2006). Isolation of Lagos bat virus from water mongoose. Emerg. Infect. Dis..

[10] Kgaladi J., Nel L.H., Markotter W. (2013). Comparison of pathogenic domains of rabies and African rabies-related lyssaviruses and pathogenicity observed in mice. Onderstepoort J. Vet. Res..

[11] Markotter W., Kuzmin I.V., Rupprecht C.E., Nel L.H. (2009). Lagos bat virus virulence in mice inoculated by the peripheral route. Epidemiol. Infect..

[12] Faber M., Pulmanausahakul R., Nagao K., Prosniak M., Rice A.B., Koprowski H., Schnell M.J., Dietzschold B. (2004). Identification of viral genomic elements responsible for rabies virus neuroinvasiveness. Proc. Natl. Acad. Sci. USA.

[13] Pulmanausahakul R., Li J., Schnell M.J., Dietzschold B. (2008). The glycoprotein and the matrix protein of rabies virus affect pathogenicity by regulating viral replication and facilitating cell-to-cell spread. J. Virol..

[14] Tao L., Ge J., Wang X., Zhai H., Hua T., Zhao B., Kong D., Yang C., Chen H., Bu Z. (2010). Molecular basis of neurovirulence of flury rabies virus vaccine strains: Importance of the polymerase and the glycoprotein R333Q mutation. J. Virol..

[15] Shimizu K., Ito N., Mita T., Yamada K., Hosokawa-Muto J., Sugiyama M., Minamoto N. (2007). Involvement of nucleoprotein, phosphoprotein, and matrix protein genes of rabies virus in virulence for adult mice. Virus Res..

[16] Dietzschold B., Schnell M.J. (2002). New approaches to the development of live attenuated rabies vaccines. Hybrid. Hybridomics..

[17] Morimoto K., Foley H.D., McGettigan J.P., Schnell M.J., Dietzschold B. (2000). Reinvestigation of the role of the rabies virus glycoprotein in viral pathogenesis using a reverse genetics approach. J. Neurovirol..

[18] Marston D.A., McElhinney L.M., Banyard A.C., Horton D.L., Núñez A., Koser M.L., Schnell M.J., Fooks A.R. (2013). Interspecies protein substitution to investigate the role of the lyssavirus glycoprotein. J. Gen. Virol..

[19] Masatani T., Ito N., Shimizu K., Ito Y., Nakagawa K., Sawaki Y., Koyama H., Sugiyama M. (2010). Rabies virus nucleoprotein functions to evade activation of the RIG-I-mediated antiviral response. J. Virol..

[20] Ito N., Takayama M., Yamada K., Sugiyama M., Minamoto N. (2001). Rescue of rabies virus from cloned cDNA and identification of the pathogenicity-related gene: Glycoprotein gene is associated with virulence for adult mice. J. Virol..

[21] Langevin C., Jaaro H., Bressanelli S., Fainzilber M., Tuffereau C. (2002). Rabies virus glycoprotein (RVG) is a trimeric ligand for the N-terminal cysteine-rich domain of the mammalian p75 neurotrophin receptor. J. Biol. Chem..

[22] Tuffereau C., Benejean J., Blondel D., Kieffer B., Flamand A. (1998). Low-affinity nerve-growth factor receptor (P75NTR) can serve as a receptor for rabies virus. EMBO J..

[23] Lentz T.L., Burrage T.G., Smith A.L., Crick J., Tignor G.H. (1982). Is the acetylcholine receptor a rabies virus receptor?. Science.

[24] Thoulouze M.-I., Lafage M., Schachner M., Hartmann U., Cremer H., Lafon M. (1998). The neural cell adhesion molecule is a receptor for rabies virus. J. Virol..

[25] Morimoto K., Hooper D.C., Spitsin S., Koprowski H., Dietzschold B. (1999). Pathogenicity of different rabies virus variants inversely correlates with apoptosis and rabies virus glycoprotein expression in infected primary neuron cultures. J. Virol..

[26] Kassis R., Larrous F., Estaquier J., Bourhy H. (2004). Lyssavirus matrix protein induces apoptosis by a TRAIL-dependent mechanism involving caspase-8 activation. J. Virol..

[27] Préhaud C., Lay S., Dietzschold B., Lafon M. (2003). Glycoprotein of nonpathogenic rabies viruses is a key determinant of human cell apoptosis. J. Virol..

[28] Genz B., Nolden T., Negatsch A., Teifke J.-P., Conzelmann K.-K., Finke S. (2012). Chimeric rabies viruses for trans-species comparison of lyssavirus glycoprotein ectodomain functions in virus replication and pathogenesis. Berl. Munch. Tierarztl. Wochenschr..

[29] Finke S., Granzow H., Hurst J., Pollin R., Mettenleiter T.C. (2010). Intergenotypic replacement of lyssavirus matrix proteins demonstrates the role of lyssavirus M proteins in intracellular virus accumulation. J. Virol..

[30] Badrane H., Bahloul C., Perrin P., Tordo N. (2001). Evidence of two Lyssavirus phylogroups with distinct pathogenicity and immunogenicity. J. Virol..

[31] Lafon M., Bourhy H., Sureau P. (1988). Immunity against the European bat rabies (Duvenhage) virus induced by rabies vaccines: An experimental study in mice. Vaccine.

[32] Malerczyk C., Selhorst T., Tordo N., Moore S., Müller T. (2009). Antibodies induced by vaccination with purified chick embryo cell culture vaccine (PCECV) cross-neutralize non-classical bat lyssavirus strains. Vaccine.

[33] Hanlon C.A., Kuzmin I.V., Blanton J.D., Weldon W.C., Manangan J.S., Rupprecht C.E. (2005). Efficacy of rabies biologics against new lyssaviruses from Eurasia. Virus Res..

[34] Jallet C., Jacob Y., Bahloul C., Drings A., Desmezieres E., Tordo N., Perrin P. (1999). Chimeric lyssavirus glycoproteins with increased immunological potential. J. Virol..

[35] Brookes S., Parsons G., Johnson N., McElhinney L., Fooks A. (2005). Rabies human diploid cell vaccine elicits cross-neutralising and cross-protecting immune responses against European and Australian bat lyssaviruses. Vaccine.

[36] Nel L.H. (2005). Vaccines for lyssaviruses other than rabies. Expert Rev. Vaccines.

[37] Horton D.L., Banyard A.C., Marston D.A., Wise E., Selden D., Nunez A., Hicks D., Lembo T., Cleaveland S., Peel A.J. (2014). Antigenic and genetic characterization of a divergent African virus, Ikoma lyssavirus. J. Gen. Virol..

[38] Kuzmin I.V., Mayer A.E., Niezgoda M., Markotter W., Agwanda B., Breiman R.F., Rupprecht C.E. (2010). Shimoni bat virus, a new representative of the *Lyssavirus* genus. Virus Res..

[39] Faber M., Pulmanausahakul R., Hodawadekar S.S., Spitsin S., McGettigan J.P., Schnell M.J., Dietzschold B. (2002). Overexpression of the rabies virus glycoprotein results in enhancement of apoptosis and antiviral immune response. J. Virol..

[40] Faber M., Li J., Kean R.B., Hooper D.C., Alugupalli K.R., Dietzschold B. (2009). Effective preexposure and postexposure prophylaxis of rabies with a highly attenuated recombinant rabies virus. Proc. Natl. Acad. Sci. USA.

[41] Hosokawa-Muto J., Ito N., Yamada K., Shimizu K., Sugiyama M., Minamoto N. (2006). Characterization of recombinant rabies virus carrying double glycoprotein genes. Microbiol. Immunol..

[42] Tao L., Ge J., Wang X., Wen Z., Zhai H., Hua T., Zhao B., Kong D., Yang C., Bu Z. (2011). Generation of a recombinant rabies Flury LEP virus carrying an additional G gene creates an improved seed virus for inactivated vaccine production. Virol. J..

[43] Cenna J., Hunter M., Tan G.S., Papaneri A.B., Ribka E.P., Schnell M.J., Marx P.A., McGettigan J.P. (2009). Replication-deficient rabies virus–based vaccines are safe and immunogenic in mice and monhuman primates. J. Infect. Dis..

[44] Faul E.J., Wanjalla C.N., McGettigan J.P., Schnell M.J. (2008). Interferon-β expressed by a rabies virus-based HIV-1 vaccine vector serves as a molecular adjuvant and decreases pathogenicity. Virology.

[45] Wen Y., Wang H., Wu H., Yang F., Tripp R.A., Hogan R.J., Fu Z.F. (2011). Rabies virus expressing dendritic cell-activating molecules enhances the innate and adaptive immune response to vaccination. J. Virol..

[46] Zhao L., Toriumi H., Wang H., Kuang Y., Guo X., Morimoto K., Fu Z.F. (2010). Expression of MIP-1α (CCL3) by a recombinant rabies virus enhances its immunogenicity by inducing innate immunity and recruiting dendritic cells and B cells. J. Virol..

[47] Pulmanausahakul R., Faber M., Morimoto K., Spitsin S., Weihe E., Hooper D.C., Schnell M.J., Dietzschold B. (2001). Overexpression of cytochrome C by a recombinant rabies virus attenuates pathogenicity and enhances antiviral immunity. J. Virol..

[48] Faber M., Faber M.-L., Li J., Preuss M.A., Schnell M.J., Dietzschold B. (2007). Dominance of a nonpathogenic glycoprotein gene over a pathogenic glycoprotein gene in rabies virus. J. Virol..

[49] Faber M., Faber M.-L., Papaneri A., Bette M., Weihe E., Dietzschold B., Schnell M.J. (2005). A single amino acid change in rabies virus glycoprotein increases virus spread and enhances virus pathogenicity. J. Virol..

[50] Schnell M.J., Mebatsion T., Conzelmann K.-K. (1994). Infectious rabies viruses from cloned cDNA. EMBO J..

[51] ProMED-mail. Rabies, bat-France. http://www.promedmail.org.

[52] Aubert F.A. Rabies in individual countries: France. http://www.who-rabiesbulletin.org/.

[53] Sato M., Maeda N., Yoshida H., Urade M., Saito S., Miyazaki T., Shibata T., Watanabe M. (1977). Plaque formation of herpes virus hominis type 2 and rubella virus in variants isolated from the colonies of BHK21/WI-2 cells formed in soft agar. Arch. Virol..

[54] Dean D.J., Abelseth M.K., Atanasiu P., Meslin F.-X., Kaplan M.M., Koprowski H. (1996). The fluorescent antibody test: p 89–85. Laboratory Techniques in Rabies.

[55] Reed L.J., Muench H. (1938). A simple method of estimating fifty per cent endpoints. Am. J. Epidemiol..

[56] Smith J.S., Yager P.A., Baer G.M., Meslin F.-X., Kaplan M.M., Koprowski H. (1996). A rapid fluorescent focus inhibition test (RFFIT) for determining rabies virus neutralizing antibody. Laboratory Techniques in Rabies.

[57] McCulloch S.D. (2013). Barcoding of South African bat species and evaluation of their natural exposure to lyssaviruses. Master’s Thesis.

[58] Mebatsion T., Schnell M.J., Conzelmann K.-K. (1995). Mokola virus glycoprotein and chimeric proteins can replace rabies virus glycoprotein in the rescue of infectious defective rabies virus particles. J. Virol..

[59] Cox J.H., Dietzschold B., Schneider L. (1977). Rabies virus glycoprotein. II. Biological and serological characterization. Infect. Immun..

[60] Dietzschold B., Wang H., Rupprecht C.E., Celis E., Tollis M., Ertl H., Heber-Katz E., Koprowski H. (1987). Induction of protective immunity against rabies by immunization with rabies virus ribonucleoprotein. Proc. Natl. Acad. Sci. USA.

[61] Mebatsion T., König M., Conzelmann K.-K. (1996). Budding of rabies virus particles in the absence of the spike glycoprotein. Cell.

[62] Etessami R., Conzelmann K.-K., Fadai-Ghotbi B., Natelson B., Tsiang H., Ceccaldi P.-E. (2000). Spread and pathogenic characteristics of a G-deficient rabies virus recombinant: An in vitro and in vivo study. J. Gen. Virol..

[63] Mebatsion T., Weiland F., Conzelmann K.-K. (1999). Matrix protein of rabies virus is responsible for the assembly and budding of bullet-shaped particles and interacts with the transmembrane spike glycoprotein G. J. Virol..

[64] Morimoto K., McGettigan J.P., Foley H.D., Hooper D.C., Dietzschold B., Schnell M.J. (2001). Genetic engineering of live rabies vaccines. Vaccine.

[65] Weyer J., Kuzmin I.V., Rupprecht C.E., Nel L.H. (2008). Cross-protective and cross-reactive immune responses to recombinant vaccinia viruses expressing full-length lyssavirus glycoprotein genes. Epidemiol. Infect..

[66] Bahloul C., Jacob Y., Tordo N., Perrin P. (1998). DNA-based immunization for exploring the enlargement of immunological cross-reactivity against the lyssaviruses. Vaccine.

[67] Dietzschold B., Kao M., Zheng Y.M., Chen Z.Y., Maul G., Fu Z.F., Rupprecht C.E., Koprowski H. (1992). Delineation of putative mechanisms involved in antibody-mediated clearance of rabies virus from the central nervous system. Proc. Natl. Acad. Sci. USA.

